# Carboxymethyl Cellulose Acetate Butyrate: A Review of the Preparations, Properties, and Applications

**DOI:** 10.1155/2014/575969

**Published:** 2014-12-04

**Authors:** Mohamed El-Sakhawy, Samir Kamel, Ahmed Salama, Hebat-Allah Sarhan

**Affiliations:** Cellulose and Paper Department, National Research Center, Dokki, Cairo 12622, Egypt

## Abstract

Carboxymethyl cellulose acetate butyrate (CMCAB) has gained increasing importance in several fields, particularly in coating technologies and pharmaceutical research. CMCAB is synthesized by esterification of CMC sodium salt with acetic and butyric anhydrides. CMCAB mixed esters are relatively high molecular weight (MW) thermoplastic polymers with high glass transition temperatures (Tg). CMCAB ester is dispersible in water and soluble in a wide range of organic solvents, allowing varied opportunity to the solvent choice. It makes application of coatings more consistent and defect-free. Its ability to slow down the release rate of highly water-soluble compounds and to increase the dissolution of poorly soluble compounds makes CMCAB a unique and potentially valuable tool in pharmaceutical and amorphous solid dispersions (ASD) formulations.

## 1. Introduction

Cellulose is the most abundant regenerated biopolymer in the planet, with annual production of about 5 × 10^11^ metric tons. Most of the cellulose is utilized in industry as a raw material in paper production. Only about 4 from 108 million tons of annually produced pulp are used for chemical production [1]. Hydroxyl groups of cellulose can be reacted to form esters or ethers of different physical and chemical properties suitable for various applications [2].

Cellulose derivatives have significant roles in industry; they represent a main source for fibers, textiles, coatings, thermoplastic films, food additives [1], and pharmaceutical technologies [3]. Cellulose derivatives are usually classified as two main classes, esters and ethers, according to the reactant nature. Cellulose derivatives usually contain free hydroxyl groups available for additional treatments to yield mixed esters. The mixed esters have several improved properties over all neat esters. Cellulose acetate propionate (CAP) and cellulose acetate butyrate (CAB) are the most commercially important mixed esters [4]. Mixed cellulose derivatives with both ester and ether groups could be also attained. Due to their low degree of substitution and high hydrolytic stability, carboxymethyl cellulose can be further esterified with organic acid anhydrides to add either single or mixed ester groups [5].

Cellulose esters in coating compositions improved many properties as hardness, aluminum flake orientation, flow and leveling, redissolving resistance, clarity, and gloss while it reduced dry-to-touch time, cratering, and blocking [6–9]. Mixed cellulose derivatives afford the benefits of conventional cellulose esters with a moderate increase in viscosity without organic solvents addition, which reduce the volatile organic compounds (VOCs) levels in coating compositions [9]. In coating compositions cellulose esters are usually applied from organic solvent solutions. Nowadays, aqueous compositions are used in adhesives, paints, and inks instead of organic solvent-based compositions in order to reduce the amount of VOC [10]. CMCAB is thought to bring health and safety benefits by replacing the organic solvent-based compositions with aqueous compositions being likely to ensue or exceed the standards performance of solvent-based compositions [11].

Amorphous solid dispersions (ASD) are smart way to improve solubility and bioavailability of drugs. CMCAB has recently shown potential as an ASD polymer [12]. CMCAB ASDs provide accelerated drug release with water-soluble drugs, while with poor water-soluble drugs it provides stable supersaturated solution, with almost zero-order release profile [13].

Cellulose derivatives containing carboxyl groups are very appropriate resources for ASD because they have excellent safety profiles, strong interactions with drug particles, the ability to adapt hydrophobicity through substituent nature and degree of substitution (DS), and high glass transition temperatures (Tg) values that promote formulation stability. High Tg preserves the medium in the glassy form even at high humidity and temperatures, regulating drug molecular motion and thus preventing crystallization during storage and transportation [12].

## 2. Preparation

Similar to the cellulose acetate preparation, CMCAB is synthesized by esterification of CMC sodium salt. CMC-Na was first protonated and reactivated with acid to transform them to the free acid form, CMC-H. Then the swollen chains are esterified with acetic and butyric anhydrides. Mixed esterification by acetic and butyric acid is taking place at the same time so; to achieve a higher DS of butyrate groups a great excess of butyric acid must be used [9, 10, 14–18]. Two commercial products were developed and marketed as a low and high MW type of this mixed ester [5].

Enzymatic acetylation of CMC by lipase provides a high selective method of mild reaction conditions with reducing the need for hazardous chemicals [19]. Heterogeneous esterification of CMC with high degree of substitution could be achieved quickly by using 4-dimethylaminopyridine or 2,2-dichloro-propionyl chloride [20].

Mostly the CMCAB esters preparation method is as follows [9, 10, 14–16].

### 2.1. Transformation of CMC-Na to the Free Acid Form

The preparation of the CMCAB is accomplished by converting CMC-Na into its acid form (CMC-H) by soaking in an aqueous sulfuric acid solution (hydrochloric acid, nitric acid, or acetic acid could also be used) and then washed with water to remove the acid.

About 100 g carboxymethyl cellulose (Na salt form, 0.30–0.65 DS carboxymethyl) was added to 2000–3000 g of 2–20% aqueous sulfuric acid at 27–60°C. The slurry was stirred for about 10–120 minutes for agitation, and the acid solution was filtered free of excess liquids and washed with demineralized water to recover CMC-H.

### 2.2. Activation of CMC (CMC-H)

The protonated CMC was transferred to a filtration funnel and excess water was drained to approximately 20–40% activated solids. The activate was dewatered by solvent exchange of the water first with three portions of acetic acid and then with three or four portions of butyric acid (each washing portion of 200–250 g acid to 100 g CMC) to give 40% solids of activated butyric acid wet CMC(H). In between each wash the sample was drained to approximately 18% solids.

### 2.3. Esterification

The butyric acid wet CMC-H was esterified by treatment with 31 g acetic anhydride and 253 g butyric anhydride at 0–15°C. A catalyst solution consisting of 3.44 g sulfuric acid in 3.44 g acetic acid was added slowly to the reaction mixture keeping the temperature below 30°C (perchloric acid, sulfoacetic acid, or zinc chloride could also be used as catalyst). After the end of the catalyst addition, the temperature was held at 30–35°C for 60–150 minutes. Then the temperature of the reaction mixture was raised to 45–60°C and held for 2–6 hours until the complete dissolution of the solids to give trisubstituted carboxymethyl cellulose, upon precipitation into water. The different acyl content of CMCAB was obtained by adjusting the ratio of acetic anhydride and butyric anhydride during esterification reaction.

Other preparing methods for CMCA suggest that, after removing the liquid well, the resultant was placed into a kneader and mixed with 250 g of acetic acid, 5.6 g of sulfuric acid, and 150 g of acetic anhydride. The reaction was performed at 48–50°C for 4 hours. Esterification could be attained also by mixing with 84 g of acetic acid, 316 g of methylene chloride, 210 g of acetic anhydride, and 1.5 g of sulfuric acid [14]. These methods could be adapted for CMCAB preparation by introducing butyric acid and butyric anhydride along/instead of acetic acid and acetic anhydride.

### 2.4. Hydrolysis and Neutralization

As with conventional esters, this ester is usually completely substituted for product uniformity. This product is then hydrolyzed using sulfuric acid to provide a desired partially substituted carboxymethyl cellulose ester. Hydrolysis is important to offer gel-free solutions in organic solvents and to provide improved compatibility with other resins in coatings formulations. The free hydroxyl groups obtained after hydrolysis are essential crosslinking sites in many coatings applications. For optimum thermal and hydrolytic stability of the final product, it is important to neutralize the strong acid catalyst [9].

A solution of 95 g water, 95 g acetic acid, and 2 g sulphuric acid was added dropwise to the reaction mixture over 30–45 minutes at 40–45°C. The contents were hydrolyzed by heating to 60–72°C for 2–4.5 hours. Then, the excess sulfuric acid is neutralized by addition of 7.53 g magnesium acetate tetrahydrate, dissolved in 25 g water and 25 g acetic acid.

### 2.5. Precipitation and Filtration

The reaction mixture, either the fully substituted or partially hydrolyzed forms of the CMCAB, was then poured into about 20 times its volume of water, and the formed precipitate was filtered, washed well from organic acids and inorganic salts with water, and dried at 60°C under vacuum to obtain the acid form of CMCAB as a white granular powder.

An optional precipitation method is to add 10% aqueous acetic in sufficient volume to yield about 30% organic acid in the final precipitation bath, about 3,000 mL, followed by the addition of an equal amount of water to harden the precipitated particles.

### 2.6. CMCAB Salts

CMCAB with DS CM of 0.3 is insoluble in water. Water solubility is achieved when the carboxylic acid groups are fully neutralized into their salts form. However, partial solubility and thus stable dispersions are attained by partial neutralization of the acid groups, where ammonia or various organic amines are commonly employed to create ammonium carboxylate salts along the polymer backbone. The tertiary amine, N,N-dimethylethanolamine, is frequently used for partial neutralization. CMCAB salts promote water interactions that stabilize the dispersion, and the degree of neutralization controls the dispersion properties. Too much neutralization will promote CMCAB water solubility, potentially resulting impractically in high dispersion/solution viscosity [6, 8].

Different CMCAB salt dispersions, sodium, potassium, calcium, or ammonium carboxylate may be formed by first dissolving CMCAB (acid form) into an organic solvent (acetone), and then 0.1–0.5 N neutralizing alkaline solution is added dropwise and then dried under vacuum by means of a rotary evaporator to give the corresponding salt.

As the CMCAB salt is obtained, the degrees of substitution by carboxymethyl group and by acetyl group were determined. The resulting white solid CMCAB had the following general composition: acid number 47–60, sulfur 25–100 PPM, DS of carboxymethyl groups 0.26–0.35, butyryl 1.64–2.26, acetyl 0.49–0.60, and hydroxyl 0.10–0.60.

## 3. Properties

Carboxymethyl cellulose acetate butyrate mixed esters are thermoplastic polymers with relatively high molecular weight (MW) and high glass transition temperatures (Tg) [16]. CMCAB is relatively nonpolar; it is insoluble in water and soluble in common organic solvents as alcohols, ketones, esters, and glycol ethers [5, 21]. By complete neutralization of the carboxylic acid groups to their salts form, water solubility occurs. CMCAB solubility is affected by many factors as carboxyl content, percent of carboxylic group neutralization, degree of substitution, substitution homogeneity, percent of acetate and butyrate groups, and the viscosity [9].

Ethyl acetate is known as a better solvent for CMCAB than acetone. CMCAB films formed from ethyl acetate solution were smooth and flat. Contact angle measurements considered CMCAB surfaces as hydrophobic due to the position of methyl groups. CMCAB surface energy is close to that of collagen [22].

Rheological properties viscosity, thixotropy loop, and dynamic viscoelasticity of CMCAB water dispersions (WD) were conducted. The viscosity of WD increases as the ratio of solvent/water decreased. Significant increase in viscosity was noticed by neutralization until a 50% degree and then it increases slowly. WD is a positive thixotropy liquid and is viscous at low concentration and neutralization degree. WD begins to form instable gel structure as concentration and neutralization degree increased [23].

## 4. Applications

Carboxymethyl cellulose acetate butyrate combines two known commercial cellulose derivatives, carboxymethyl cellulose (CMC) and cellulose acetate butyrate (CAB). CMCAB is specifically designed for use in the waterborne coating industry. CMCAB makes coatings application more consistent and defect free; it provides to waterborne systems improved flow and leveling combined with sag resistance, reduces dry time, controls viscosity, has excellent metal flake control, and also facilitates the dispersion of hydrophobic species into water-based coating systems [24]. CMCAB attains these properties through high Tg, near-Newtonian rheology, and a sharp viscosity/solids relationship [6].

The aqueous pigment dispersions could be prepared by adding an organic solvent solution of the CMCAB ester to a metallic dispersion and neutralizing, partially or fully, the ester by base addition (ammonia or amine) and then mixing in water. The resulting mixture may then be added directly to a dispersion containing only aluminum flake and an organic solvent. The esters also have specific use as wetting agents in high solids coatings [16]. Low molecular weight carboxyalkyl cellulose esters are useful in coating and ink compositions as low viscosity binder resins and rheology modifiers [9].

A stable aqueous coating dispersion is provided containing up to 50%, based on the total solids weight, cellulose mixed ester with organic solvent, water, and a suitable amine neutralized acrylic resin [25, 26]. Aqueous dispersion of carboxylated cellulose esters in water that is suitable for use in a variety of waterborne coating compositions while having a relatively low VOC content was suggested [8].

Low molecular weight CMCAB with a maximum degree of substitution as coating additive provides high solids and low viscosity coating compositions, without the drawbacks usually found with typical low molecular weight cellulose esters such as the increase in organic solvent ratio to reserve the required viscosity. They have a marginal effect on solution and spray viscosities of high solids coatings. It displays superior compatibility when mixed with other coating resins, thus yielding clear films compared to conventional cellulose esters [9].

CMCAB provides enhanced aluminum flake orientation and improved hardness. They can provide a high gloss protective coating for several substrates, especially metal and wood. The solids percent can be increased relative to organic solvent, consequently reducing the VOC of the formulation [9].

When carboxymethyl cellulose esters of higher acids, including CMCAB, are treated with ammonia or an amine they are readily dispersed in waterborne formulations, containing metallic pigments such as aluminum flakes and mica [16]. It improves rheological properties by a remarkable increase in viscosity with a minor increase in ester concentration [15, 27].

To prevent sagging and dripping in waterborne coatings, rapid solids increases due to solvent evaporation are important. Due to its exceptional balance of hydrophilicity from the carboxylate groups and hydrophobicity from its acetate groups, CMCAB affords a good substrate wetting with wonderful flow and leveling and excellent redissolving resistance and decreased defects in waterborne paints as cratering, sagging, and picture framing [6]. Also CMCAB in water dispersions keeps metallic flakes orientation in suspension considerably longer compared with polyurethane thickeners [7]. CMCAB has high compatibility with common plasticizers used in coating applications so clear films were produced compared to conventional cellulose esters [10].

High solids and water dispersions of CMCAB mixed esters are used as renewable wood adhesives [5]. Aqueous CMCAB size composition improves the holdout of a coated, cellulosic fiber board [10]. Holdout is the ability of a coating to stay at or near the substrate surface, not to penetrate that substrate. As a result of enhanced holdout the coated surface appears smoother, uniform with increased gloss [10]. CMCAB in a wood stain formulation provides good adhesion under an overcoat [9].

As a wood adhesive, CMCAB dispersions will require higher solids contents up to 40%, such that the resulting films would create an adhesive layer with mechanical integrity. Adhesive viscosity, film formation, penetration, joint-performance, and fracture toughness are dependent on MW, the percent solids, percent neutralization, and the organic solvent components. CMCAB interacts strongly with wood and provides a potential use as wood adhesive [5].

Although petroleum glassy polymers have a huge market, there are few studies on glassy polymers from biomass [28]. Amorphous glassy polymers, like CMCAB, may produce strong films. The glass transition temperature (Tg) is the temperature at which amorphous polymers soften and attain mobility like viscous liquid [29]. Polymers of higher Tg values produced frequently brittle films and need plasticizers to improve their properties [30]. Usually esters with smaller substituents, such as CA, have higher Tgs, roughly 180°C, and hence require more plasticizer than mixed esters with larger groups like CMCAB with a Tg between 135 and 141°C [21, 31]. CMCAB, with its relatively lower Tg than many cellulose esters, is an easily compressed matrix for pharmaceutical actives. When used in proper amounts, it is possible to produce a CMCAB/drug tablet for a one-time dose to last up to 24 h. CMCAB matrix provides pH controlled release with no need for an enteric coating of the tablet [21].

CMCAB has good miscibility with various pharmaceutical actives. It forms amorphous matrices with poorly soluble drugs and enhances its water solubility and bioavailability [21]. CMCAB in drugs formulation provides slow or zero-order pH controlled release, stable solid blends, and better drug solubility and stabilization [12]. CMCAB was compressed with drugs into tablets to get the pH-responsive drug delivery systems [21].

Amorphous solid dispersions (ASD) are a smart technique to increase the drugs water solubility for oral bioavailability. Carboxylated cellulose derivatives are very appropriate composition for ASD due to their unique properties of no toxicity and high Tg and they provide strong hydrogen bonding with the drug to create pH controlled drug release. CMCAB is functional ASD to avoid drugs crystallization, while controlling drug release to the pH of the small intestine [31].

Carboxylated cellulose derivatives were evaluated for their ability to form an ASD with ellagic acid (bioactive natural flavonoid) [32], quercetin (dietary flavonoid abundant in foods) [33], curcumin (hydrophobic polyphenol) [34], and naringenin (bioactive flavonoid) [13] in order to improve their solubility in aqueous solution. These compounds and cellulose esters were readily blended by spray-drying to produce amorphous solid dispersions even at very high concentrations. ASD is an interesting approach to enhance solubility, bioavailability, effectiveness, simplicity, benign nature, and quite slow release from CMCAB dispersions.

Poly(3-hydroxybutyrate) (PHB) gel formation blends with CMCAB were prepared. The gel formation procedure provided new ways to prepare immiscible blends with the advantage of using benign solvents [35].

Nanofibrous CMCAB mats with excellent superhydrophobicity were prepared by electrospinning [36]. Spin-coated films were prepared from CMCAB solution in tetrahydrofuran (THF). THF vapor allowed the cellulose esters chains mobility, causing considerable changes in the film morphology. Smooth and homogeneous porous CMCAB films were detected after only 3 min of exposure to THF vapor [37].

CMCAB is expected to have the same or superior benefit provided by carboxymethyl cellulose acetate (CMCA). CMCA, in membranes for reverse osmosis, give an elastic film that was tougher and of better desalination characteristics than the standard cellulose acetate membrane [38]. Blending of CMCA/cellulose acetate (CA) resulted in novel blend membranes with enhanced features such as lower contact angle. Morphology, permeability, hydrophilicity, and antifouling properties of the prepared CA/CMCA blend membranes enhanced considerably by the combination of CMCA and CA [17].

Acetylated cellulose ether provides advancing properties and bleach activation to the heavy duty detergent composition. The acetylated ether acts as a bleach activator by reacting with a bleaching agent, such as sodium perborate monohydrate, to generate peracetic acid [39].

Carbohydrate based surfactants are largely used in microemulsions and in drugs solubilization. Thin films of mixtures containing CMCAB and carbohydrate based surfactant, sorbitanmonopalmitate or poly(oxyethylene) sorbitanmonopalmitate, were spin-coated onto silicon wafers [31]. CMCAB films were deposited from ethyl acetate solutions onto silicon wafers or aminoterminated surfaces and used for lectins adsorption [40].

Nanometer fiber composite material was obtained from lithium iron phosphate (LiFePO4, LFP)/CMCAB by electrospinning. Under the protection of inert gas, modified LFP/carbon nanofibers (CNF) were achieved by carbonization in 600°C. Cellulose materials that were applied to lithium battery by electrospinning can improve battery performance [18]. CMCAB had a structure of multicarbon functional groups, which provide abundant carbon resources and attain a dense conductive carbon structure by modification. This property established the CMCAB application in the electrochemistry [18].

## References

[1] Qiu X., Hu S. (2013). ‘Smart’ materials based on cellulose: a review of the preparations, properties, and applications. *Materials*.

[2] Shanbhag A., Barclay B., Koziara J., Shivanand P. (2007). Application of cellulose acetate butyrate-based membrane for osmotic drug delivery. *Cellulose*.

[3] Shokri J., Adibkia K. (2013). Cellulose-medical, pharmaceutical and electronic applications, chapter 3, application of cellulose and cellulose derivatives. *Pharmaceutical Industries*.

[4] Balser K., Hoppe L., Eicher T., Wandel M., Astheimer H.-J., Steinmeier H., Allen J. M., Bohnet M., Bellussi G., Bus J., Cornils B., Drauz K. (2004). Cellulose esters. *Ullmann's Encyclopedia of Industrial Chemistry*.

[5] Paris J. L. (2010). *Carboxymethyl cellulose acetate butyrate water-dispersions as renewable wood adhesives [M.S. thesis]*.

[6] Lawniczak J. E., Posey-Dowty J., Seo K. S., Walker K. (2003). Rheological aspects of carboxymethyl cellulose acetate butyrate (CMCABTM) in waterborne coatings. *Paint and Coatings Industry*.

[7] Posey-Dowty J. D., Seo K. S., Walker K. R., Wilson A. K. (2002). Carboxymethylcellulose acetate butyrate in water-based automotive paints. *Surface Coatings International Part B: Coatings International*.

[8] McCreight K. W., Webster D. C., Kemp L. K. Aqueous dispersions of carboxylated cellulose esters, and methods of making them.

[9] Shelton M. C., Wilson A. K., Posey-Dowty J. D., Kramer G. A., Perdomo L. G. R. (2011). Low molecular weight carboxyalkyl cellulose esters and their use as low viscosity binders and modifiers in coating compositions. *Patent*.

[10] Obie R. (2006). Use of carboxymethyl cellulose acetate butyrate as a precoat or size for cellulosic man-made fiber boards. *US Patent No. 0090462, US 7026470*.

[11] Obie R. T. Use of carboxyalkyl cellulose esters, such as carboxymethyl cellulose acetate butyrate, to form aqueous dispersions of hydrophobic materials in water.

[12] Li M. C., Posey-Dowty J. D., Lingerfelt L. R., Kirk S. K., Klein S., Edgar K. J., Edgar K. J., Heinze T., Liebert T. Enhanced dissolution of poorly soluble drugs from solid dispersions in carboxymethyl cellulose acetate butyrate matrices. *Polysaccharide Materials: Performance by Design*.

[13] Li B., Liu H., Amin M., Wegiel L. A., Taylor L. S., Edgar K. J. (2013). Enhancement of naringenin solution concentration by solid dispersion in cellulose derivative matrices. *Cellulose*.

[14] Namikoshi H. Carboxyalkyl acetyl celluloses, Their salts and a process for the preparation of them.

[15] Allen J. M., Wilson A. K., Lucas P. L., Curtis L. G. (1998). Process for preparing carboxyalkyl cellulose esters. *Patent*.

[16] Posey-Dowty J. D., Wilson A. K., Curtis L. G., Swan P. M., Seo K. S. Carboxyallkyl cellulose esters for use in aqueous pigment dispersions.

[17] Han B., Zhang D., Shao Z., Kong L., Lv S. (2013). Preparation and characterization of cellulose acetate/carboxymethyl cellulose acetate blend ultrafiltration membranes. *Desalination*.

[18] Qiu L., Shao Z., Yang M., Wang W., Wang F., Xie L., Lv S., Zhang Y. (2013). Electrospun carboxymethyl cellulose acetate butyrate (CMCAB) nanofiber for high rate lithium-ion battery. *Carbohydrate Polymers*.

[19] Wang Y.-J., Yang K. Enzymatic acetylation of carboxymethyl cellulose by lipases.

[20] Klemm D., Heinze T. (1986). Acylation of carboxymethyl cellulose accelerated by 4-dimethylaminopyridine. *Synthetic Communications*.

[21] Posey-Dowty J. D., Watterson T. L., Wilson A. K., Edgar K. J., Shelton M. C., Lingerfelt L. R. (2007). Zero-order release formulations using a novel cellulose ester. *Cellulose*.

[22] Amim J., Petri D. F. S., Maia F. C. B., Miranda P. B. (2009). Solution behavior and surface properties of carboxymethyl cellulose acetate butyrate. *Cellulose*.

[23] Lv S.-Y., Shao Z.-Q., Wang F.-J., Wang W.-J., Li Y.-H. (2010). Rheological properties of carboxymethyl cellulose acetate butyrate water dispersions. *Polymeric Materials Science and Engineering*.

[24] Eggers D., Dillard L. R. Recent developments for carboxymethyl cellulose acetate butyrate in waterborne coatings.

[25] Walker K. R. Aqueous coatings composition containing cellulose mixed ester and amine neutralized acrylic resin and the process for the preparation thereof.

[26] Kuo C. M., Curtis L. G., Lucas P. L. (1994). Aqueous dispersion useful in coatings containing hydrolyzed cellulose ester and acrylic resin. *Patent No.*.

[27] Allen J. M., Wilson A. K., Lucas P. L., Curtis L. G. Carboxyalkyl cellulose esters.

[28] Yamaguchi M., Masuzawa K. (2008). Transparent polymer blends composed of cellulose acetate propionate and poly(epichlorohydrin). *Cellulose*.

[29] Wiese H., Urban D., Takamura K. (2002). Characterization of aqueous polymer dispersions. *Polymer Dispersions and their Industrial Applications*.

[30] Wadey B. L., Robert A. M. (2001). Plasticizers. *Encyclopedia of Physical Science and Technology*.

[31] Amim J., Kawano Y., Petri D. F. S. (2009). Thin films of carbohydrate based surfactants and carboxymethyl cellulose acetate butyrate mixtures: morphology and thermal behavior. *Materials Science and Engineering C*.

[32] Li B., Harich K., Wegiel L., Taylor L. S., Edgar K. J. (2013). Stability and solubility enhancement of ellagic acid in cellulose ester solid dispersions. *Carbohydrate Polymers*.

[33] Li B., Konecke S., Harich K., Wegiel L., Taylor L. S., Edgar K. J. (2013). Solid dispersion of quercetin in cellulose derivative matrices influences both solubility and stability. *Carbohydrate Polymers*.

[34] Li B., Konecke S., Wegiel L. A., Taylor L. S., Edgar K. J. (2013). Both solubility and chemical stability of curcumin are enhanced by solid dispersion in cellulose derivative matrices. *Carbohydrate Polymers*.

[35] Gomes A. L., Rodrigues G. V., Goncalves M. C. Preparation of poly (3-hydroxybutyrate)/carboxymethyl cellulose acetate butyrate blends using gel formation.

[36] Xie L., Shao Z., Wang W., Wang F. (2012). Superhydrophobicity of CMCAB fibrous mats produced by electrospinning. *Integrated Ferroelectrics*.

[37] Blachechen L. S., Souza M. A., Petri D. F. S. (2012). Effect of humidity and solvent vapor phase on cellulose esters films. *Cellulose*.

[38] El-Taraboulsi M. A., Mandil M. A., Ali H. E.-S. M. (1970). Reverse osmosis studies on desalination membranes formed from chemically modified cellulose acetate. *Carbohydrate Research*.

[39] Broze G. (1989). Acetylated sugar ethers as bleach activators and detergency boosters. *Patent No.*.

[40] Amim J., Petri D. F. S. (2012). Effect of amino-terminated substrates onto surface properties of cellulose esters and their interaction with lectins. *Materials Science and Engineering C*.

